# Supplementary material for: A study protocol for comparing the treatment of varicose tributaries either concomitantly with or separately from endovenous laser ablation of the incompetent saphenous trunk (the FinnTrunk Study). A multicenter parallel-group randomized controlled study
Source: PLoS One. 2023 May 23;18(5):e0285823. doi: 10.1371/journal.pone.0285823 (PMC10204998; doi:10.1371/journal.pone.0285823)

Turku University Hospital/Khalil Firoozi, Lic.Med.
31 August 2020

Version 1.01.01

Endovenous Laser Ablation (EVLA): Significance and Timing of Varicose Tributary Treatment

1. Introduction

Endovenous methods have become well-established in the treatment of venous insufficiency. Thermal ablation – laser ablation (EVLA) or radiofrequency ablation (RFA) – under tumescence anesthesia is now a standard method for treating the main trunk of an incompetent superficial vein. Recurrence of venous insufficiency has been shown to be rarer after thermal ablation than after traditional open surgery. Furthermore, the outcomes of thermal ablation in terms of quality of life scores have been found to be equally good or better compared with open surgery.

There are differing points of view on treating the tributary branches of varicose veins. In some treatment centers, insufficiency of a superficial main trunk is treated by thermal ablation during the initial stage, and varicose tributaries are treated later at a separate visit as needed. In others, tributaries are treated along with the main trunk as part of the same procedure. Studies on the timing of tributary treatment are heterogeneous and based on small cohorts. The significance of remaining varicose tributaries (the varicose reservoir) as a possible risk factor for disease recurrence is not known.

2. Objectives

The study aims to determine whether the timing of varicose tributary treatment is significant in terms of treatment outcome or recurrence risk, as well as in terms of post-procedural pain and a patient's quality of life. Another goal is to investigate treatment cost-effectiveness, as well as how many patients later require procedures on the tributaries if only the main trunk of an incompetent superficial vein was treated during the initial stage.

The study objectives are:

- To determine whether the timing of tributary treatment is significant in terms of varicosis treatment and recurrence risk.

- To investigate patient satisfaction in patients who only have the main trunk of an incompetent superficial vein treated by laser ablation as compared with those who have varicose tributaries treated by foam sclerotherapy in conjunction with the main procedure.

- To investigate treatment cost-effectiveness as well as how many patients later require treatment of the tributaries if only the main trunk of an incompetent vein was treated during the initial stage.

- To determine how many of the patients whose tributaries were treated at the time of the main trunk procedure require additional procedures later. Another objective is to determine how significant varicose tributaries are in terms of venous insufficiency recurrence.

3. Study materials and methods

This is a randomized, controlled, multi-center study to be conducted in Finland. The study will recruit subjects who have not previously had venous interventions and who have been diagnosed with primary insufficiency of a superficial main trunk without deep venous insufficiency (CEAP classification $C_{2-3}E_pA_sP_r$). The exclusion criteria for the study are uncontrolled heart failure, limb swelling clinically presumed to be primarily due to insufficient lymph circulation, and significant occlusive arterial disease of the lower limbs.

After written consent is obtained, subjects will be randomized into one of two treatment groups. In both groups, the incompetent superficial main trunk will be treated by thermal ablation. In the first group, varicose tributaries will not be treated in conjunction with this. In the second group, the tributaries will be treated by foam sclerotherapy. Treatment outcomes in both groups will be reviewed on follow-up three months after the procedure, at which time remaining varicose tributaries will be treated by foam sclerotherapy as needed. Further follow-up of treatment outcomes will take place at 1, 3, and 5 years (ultrasound and clinical assessment).

The study endpoints are: treatment time, need for tributary treatment after the initial procedure, post-procedural pain (VAS scale and need for pain medications), quality of treatment based on the Aberdeen Varicose Vein Questionnaire (AVVQ) score for venous insufficiency treatment, and procedural complications.

Study register BCB/HUS eCRF, other?

Turku University Hospital/Khalil Firoozi, Lic.Med.
31 August 2020

Randomization

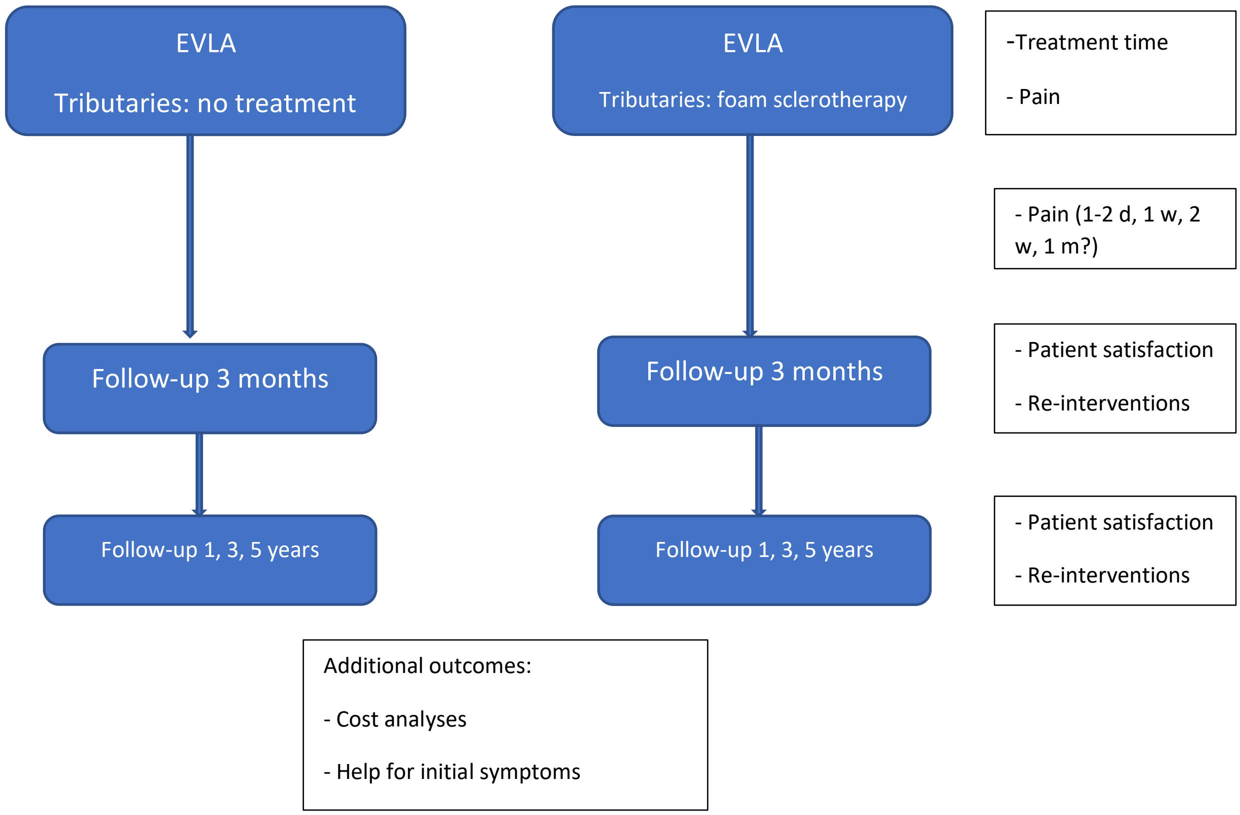

Supplement: S1 File — (PDF) [file pone.0285823.s002.pdf]
