# Supplementary material for: A study protocol for comparing the treatment of varicose tributaries either concomitantly with or separately from endovenous laser ablation of the incompetent saphenous trunk (the FinnTrunk Study). A multicenter parallel-group randomized controlled study
Source: PLoS One. 2023 May 23;18(5):e0285823. doi: 10.1371/journal.pone.0285823 (PMC10204998; doi:10.1371/journal.pone.0285823)
Supplement: S2 File — (DOCX) [file pone.0285823.s003.docx]

Versio 1.01.01

Laskimonsisäinen laserablaatio (EVLA): kohjuuntuneiden sivuhaarojen hoidon merkitys ja hoidon ajoitus

# Johdanto

Laskimonsisäiset menetelmät ovat vakiinnuttaneet asemansa laskimovajaatoiminnan hoidossa, ja vajaatoimintaisen pinnallisen päärungon hoidon standardimenetelmä on nykyisin tumesenssipuudutuksessa tehtävä termoablaatio (laserablaatio EVLA tai radiotaajuusablaatio RFA). Laskimovajaatoiminnan uusiutumisen on termoablaation jälkeen osoitettu olevan perinteistä avoleikkausta harvinaisempaa, minkä lisäksi termoablaation tulokset ovat avoleikkaukseen verrattuina yhtä hyviä tai parempia myös elämänlaatumittareilla (quality of life score) tarkasteltaessa.

Kohjuuntuneiden sivuhaarojen hoidosta on useita näkemyksiä. Osassa keskuksista pinnallisen päärungon vajaatoiminta hoidetaan ensimmäisessä vaiheessa termoablaatiolla ja kohjuuntuneet sivuhaarat sen jälkeen tarvittaessa erillisellä käynnillä, osassa keskuksista sivuhaarat hoidetaan päärunkotoimenpiteen yhteydessä. Tutkimukset sivuhaarojen hoidon ajoituksesta ovat heterogeenisiä ja perustuvat pieniin kohortteihin. Jäljellä olevien kohjuuntuneiden sivuhaarojen (varicose reservoir) merkitystä mahdollisena taudin uusimisille altistavana tekijänä ei tunneta.

# Tavoitteet

Tutkimuksen tavoitteena on selvittää, onko kohjuuntuneiden sivuhaarojen hoidon ajoituksella merkitystä hoitotuloksen tai taudin uusiutumisriskin kannalta, ja onko hoidon ajoituksella toisaalta merkitystä toimenpiteen jälkeisen kivun ja potilaan elämänlaadun kannalta. Tavoitteena on myös tarkastella hoidon kustannustehokkuutta sekä sitä, kuinka moni potilaista tarvitsee myöhemmin sivuhaarojen toimenpiteitä, jos ensimmäisessä vaiheessa on hoidettu vain vajaatoimintainen pinnallinen päärunko.

Tutkimuksen tavoitteet ovat:

-Selvittää, onko sivuhaarojen hoidon ajoituksella merkitystä taudin hoidon ja uusiutumisriskin kannalta.

-Selvittää potilastyytyväisyyttä potilailta, joilta hoidetaan vain pinnallisen päärungon vajaatoiminta laserablaatiolla ja verrata näitä potilaita ryhmään, jossa kohjuuntuneet sivuhaarat hoidetaan vaahtoskleroterapialla päärungon toimenpiteen yhteydessä.

-Tarkastella hoidon kustannustehokkuutta sekä sitä, kuinka moni potilaista tarvitsee myöhemmin sivuhaarojen hoitoa, jos ensimmäisessä vaiheessa on hoidettu vain vajaatoimintainen päärunko.

-Selvitetään, kuinka moni niistä potilaista, joilta sivuhaarat on hoidettu päärungon toimenpiteen yhteydessä, tarvitsee myöhemmin lisätoimenpiteitä. Tavoitteena on myös selvittää, mikä merkitys kohjuuntuneilla sivuhaaroilla on laskimovajaatoiminnan uusimisen kannalta.

# Tutkimusaineisto ja -menetelmät

Tutkimus on satunnaistettu,vertaileva kansallinen monikeskustutkimus. Tutkimukseen rekrytoidaan tutkittavia, joille ei ole aiemmin tehty laskimotoimenpiteitä, ja joilta on todettu pinnallisen päärungon primaarinen vajaatoiminta ilman syvien laskimoiden vajaatoimintaa (CEAP-luokituksessa C_2-3_E_p_A_s_P_r_)_._ Tutkimuksen poissulkukriteerit ovat hoitamaton sydämen vajaatoiminta, kliinisesti ensisijaisessti imunestekierron vajauksesta johtuvaksi oletettu raajan turvotus ja merkittävä alaraajojen tukkiva valtimotauti.

Tutkittavat satunnaistetaan kirjallisen suostumuksen jälkeen jompaankumpaan kahdesta käsittelyryhmästä. Vajaatoimintainen pinnallinen päärunko hoidetaan kummassakin ryhmässä termoablaatiolla. Ensimmäisessä ryhmässä kohjuuntuneita sivuhaaroja ei hoideta tässä yhteydessä, ja toisessa ryhmässä sivuhaarat hoidetaan vaahtoskleroterapilalla. Hoitotulos kontrolloidaan kummassakin ryhmässä 3 kuukauden kuluttua toimenpiteen jälkeen, jolloin jäljellä olevat kohjuuntuneet sivuhaarat hoidetaan tarvittaessa vaahtoskleroterapialla. Tämän jälkeen hoitotulos kontrolloidaan 1, 3 ja 5 vuoden kohdalla (ultraääni ja kliininen arvio).

Tutkimuksen päätemuuttujat ovat: toimenpideaika, sivuhaarojen hoidon tarve ensimmäisen toimenpiteen jälkeen, toimenpiteen jälkeinen kipu (VAS mittari ja kipulääkkeen tarve), hoidon laatu laskimovajaatoiminnan hoitoon suunnitellulla Aberdeen Varicose Vein Questionnaire (AVVQ) scorella ja toimenpiteeseen liittyvät komplikaatiot.

Tutkimusrekisteri BCB/HUS eCRF, joku muu?

Randomization

-Patient satisfaction

-Re-interventions

-Truncal reflux

Control 1, 3, 5 years

Control 1, 3, 5 years

-Pain (1-2 d, 1 w, 2 w, 1 m?)

-Patient satisfaction

-Re-interventions

-Truncal reflux

Control 3 months

Control 3 months

-Treatment time

-Pain

-Patient satisfaction

EVLA

Tributaries foam sclerotherapy

EVLA

Tributaries no treatment

Additional outcomes:

-Cost analyses

-Help for initial symptoms

-Requirement for re-interventions
