# Supplementary material for: A study protocol for comparing the treatment of varicose tributaries either concomitantly with or separately from endovenous laser ablation of the incompetent saphenous trunk (the FinnTrunk Study). A multicenter parallel-group randomized controlled study
Source: PLoS One. 2023 May 23;18(5):e0285823. doi: 10.1371/journal.pone.0285823 (PMC10204998; doi:10.1371/journal.pone.0285823)
Supplement: Attachment — Submitted filename: Comments of Finn Trunk.docx [file pone.0285823.s004.docx]

General comments:

I am honored to reviewe Dr. Harri Hakovirta’s manuscript entitled "A study protocol for comparing the treatment of varicose tributaries either concomitantly with or separately from endovenous laser ablation of the incompetent saphenous trunk (the FinnTrunk Study). A multicenter parallel-group randomized controlled study”. Overall, this is a good study, however, this study have obvious selective bias in the study setting, which might affect the This may affect the objectivity and generalizability of the conclusions.

1. Page 3 line 51-67: as you mentioned in this section, varicose tributaries in VVs patients are very common, even all patients have the tributaries insuffiency, thus nowadays guideline recommend the combined approach for VVs; and the UGFS may accompany with the high recurrence and lower closure rate, thus, the group setting in this study may occur some selective bias. Please revise.
2. Page 2 line 29 and page 5-6 line 105-108 and Table 2: in this study, the author only collect the patients with CEAP classification C2-3, obviously, the selective criteria display some bias, because there are a lot of patients have the C4-5, therefore, this study setting will lose some very important patients, and then the conclusion might be affected. I suggest that the authors make modifications to the selection criteria.
3. Page 7 line 140-144: Many patients with stage C2 have no obvious symptoms, but only show varicose veins. How the author screens out symptomatic patients from a large number of stage C2 patients is a huge workload. Please explain.
4. Page 10 line 229 interventions. In this study the author uses the endovenous laser as the thermal procedure, why don’t you use the radiofrequency ablation; what is more, the author need to clarify whether thermal ablation is only used for the upper knee GSV trunk or the entire GSV, which is very important for the study.
5. In this study, the author did not discuss the setting, advantages and disadvantages of the study, especially with other similar studies. This part needs to be supplemented.
