# Supplementary material for: A study protocol for comparing the treatment of varicose tributaries either concomitantly with or separately from endovenous laser ablation of the incompetent saphenous trunk (the FinnTrunk Study). A multicenter parallel-group randomized controlled study
Source: PLoS One. 2023 May 23;18(5):e0285823. doi: 10.1371/journal.pone.0285823 (PMC10204998; doi:10.1371/journal.pone.0285823)
Supplement: Attachment — Submitted filename: renamed_27975.docx [file pone.0285823.s005.docx]

# COMMENTS – Manuscript PONE-D-23-03544

Title: “A study protocol for comparing the treatment of varicose tributaries either concomitantly with or separately from endovenous laser ablation of the incompetent saphenous trunk (FinnTrunk Study). A multicenter parallel-group randomized controlled study”

**Important note: This review pertains only to ‘statistical aspects’ of the study and so ‘clinical aspects’ [like medical importance, relevance of the study, ‘clinical significance and implication(s)’ of the whole study, etc.] are to be evaluated [should be assessed] separately/independently. Further please note that any ‘statistical review’ is generally done under the assumption that (such) study specific methodological [as well as execution] issues are perfectly taken care of by the investigator(s). This review is not an exception to that and so does not cover clinical aspects {however, seldom comments are made only if those issues are intimately / scientifically related & intermingle with ‘statistical aspects’ of the study}. Agreed that ‘statistical methods’ are used as just tools here, however, they are vital part of methodology [and so should be given due importance]. I look at the manuscript in/with statistical view point, other reviewer(s) look(s) at it with different angle so that in totality the review is very comprehensive. However, there should be efforts from authors side to improve (may be by taking clues from reviewer’s comments). Therefore, please do not limit the revision only (with respect) to comments made here.**

**COMMENTS:** I wonder, if the title can be little shorter [say as:”A study protocol for comparing the treatment of varicose disease: A multicenter parallel-group randomized controlled study“ because the aim (as per lines 83-84) is “The aim of the study is to compare two different treatment strategies for varicose disease”]? More specifically, the aim is described in lines 84 onwards anyway.

At few places correction in ‘English’ [sentence construction] is needed. For example: [only 2 are given]

Lines 31-32: Patients will be followed-up at 3 months, 1 year, 3 years, and 5 years.

Possible correction suggested: Patients will be followed-up for 5 years with evaluation recorded at 3 months, 1 year, 3 years, and 5 years [called follow-up visits]. {correct sentence in line 261: The duration of the follow-up will be five years.}

Lines 321-322: Mean values and standard deviations (SD) will be applied for normal distributions.

Possible correction suggested: Mean values and standard deviations (SD) will be estimated (or reported/displayed/tabulated) for variables having normal distributions. {note that we never say: we have apply Mean values and standard deviations (SD)}

Kindly check for the ‘English’ language. Agreed that English is not (may not be) our mother tongue however, remember/mind you that this is a scientific/academic document and so all details should be clearly/correctly communicated (do not take readers’ for granted). You may take help of language professional expert, if needed.

Is the sentence/statement “The first study results will be published in a peer reviewed journal in 2024.” of lines 79-80 necessary? What purpose it serves? Account given (both description & action indicated) in lines 152 to 157 is not understood. This phenomenon (confusing description) is/was, unfortunately, observed/noted at few more places.

Because you stated in lines 323-325 that “the Student’s t-test and ANOVA will be used to compare continuous variables of normal distributions of the values that have previously been tested using the Shapiro–Wilk test.”, please note though the measures/tools used are appropriate [ex. Aberdeen Varicose Vein Questionnaire (AVVQ) score, the Venous Clinical Severity Score 266 (VCSS), health-related quality of life (the EuroQoL EQ-5D-5L) score, pain score based on numeric rating scale (NRS)], most of them are likely to yield data that are in ‘ordinal’ level of measurement [and not in ratio level of measurement for sure {as the score two times higher does not indicate presence of that parameter/phenomenon as double (for example, a Visual Analogue Scales VAS score or say ‘depression’ score)}]. Then application of suitable non-parametric test(s) is/are indicated/advisable [even if distribution may be ‘Gaussian’ (also called ‘normal’)]. Agreed that there is/are no non-parametric test(s)/technique(s) available to be used as alternative in all situation(s) [suitable / most desired/applicable], but should be used whenever/wherever they are available. Therefore, in short use suitable non-parametric test(s)/technique(s) while dealing with data that are in ‘ordinal’ level of measurement even if [despite that] the distribution may be ‘Gaussian’. Testing ‘normality’ in sample [by using any normality test(s)} is not required/desired while dealing with data that are in ‘ordinal’ level of measurement [as most of the normality tests are not valid for ‘ordinal’ data (including Shapiro–Wilk test)].

I suggest authors to check and re-draft the ‘Conclusions’ section (lines 328 to 334). Most of these are would/could not be part of this study. In my opinion, this study has potentials but to rescue this article (which is quite possible), some amount of re-vision (re-drafting) may be needed. However, please do not limit the revision only (with respect) to comments made here. More improvement is expected.

As pointed out in ‘important note’ above “This review pertains only to ‘statistical aspects’ of the study and so ‘clinical aspects’ should be assessed separately/independently [one should carefully consider/look at the clinical implications of the study]. The respected ‘Editor’ may consider accepting only if found ‘clinical implications’ valuable (add to clinical knowledge / positively influence clinical practice). ‘Major revision’ is recommended.
